# Comparison of the Rey Auditory Verbal Learning Test (RAVLT) and Digit Test among Typically Achieving and Gifted Students

**Published:** 2016

**Authors:** Elham KHOSRAVI FARD, Jennifer L. KEELOR, Alireza Akbarzadeh BAGHEBAN, Robert W. KEITH

**Affiliations:** 1 Audiologist, ENT Unit, Sajjad Hospital, Bahar Ave, Hafte tir Square, Tehran, Iran; 2 Master of Arts, Communication Sciences and Disorders, University of Cincinnati, Cincinnati, Ohio, Canada; 3 Department of Biostatisticsm, Faculty of Paramedical Sciences, Shahid Beheshti University of Medical Sciences, Tehran, Iran; 4 Audiologist, Communication Sciences andDisorders, University of Cincinnati,Cincinnati, Ohio, Canada

## Abstract

**Objective:**

In this study, different kinds of memory were evaluated using Rey Auditory Verbal Learning (RAVLT) test and were compared between two groups of typical and gifted students using Digit Span test. Finally, we determined if working memory interfered with scores in different Rey stages or not.

**Material & Methods:**

This study was conducted in Tehran City, Iran in 2013. Scores on RAVLT were compared with WISC- R digit span results in a sample of 148 male students aged 12-14 yr old divided into two groups including 75 students in typical school (IQ ranging between 90 and 110) and 73 gifted students (IQs ranging between 110 and 130).

**Results:**

Gifted students obtained higher scores than typical students in both Forward Digit Span (FDS) and Backward Digit Span (BDS) and all 9 stages of RAVLT comparing with typical students (P<0.001). There was no significant difference between different ages (P> 0.05). The 14 yr old students in both groups had the highest score. There was a high correlation between FDS and the first stage of RAVLT as well as high correlation between BDS and seventh stage of RAVLT.

**Conclusion:**

Intelligence has effect on better score of memory and gifted subjects had better scores in memory tests, although the intelligence effect in learning was quantitative rather than qualitative. RAVLT is a comprehensive test, which evaluates short-term memory, working memory and long-term memory and besides Digit span test provides precious information about memory and learning of subjects in order to program different student’s educational schedules.

## Introduction

Learning, a change in behavior due to experience, is a key factor in language development (1). Some factors such as “intelligence” and “memory” have essential effects on learning but how they affect learning is controversial (2, 3). One unavoidable fact is that mental abilities such as intelligence and memory cannot be considered separately because they are intertwined and both of them can lead subjects to improve their academic achievement. Intelligence is a key factor for learning and in spite of the long history of studies (4, 5), there is no specific definition for it.

One common accepted definition described by Wechsler (4) is: “Intelligence is an individual’s ability to adapt and constructively solve problems in the environment.

Indexes in Wechsler definition are as follows: Verbal Comprehension, Perceptual Reasoning, Working Memory (WM) and Processing Speed. According to Wechsler, WM as will be described below is an important component of assessing intelligence.

On the other hand, memory is a relatively permanent recording of an experience, fundamental for learning.

It is classified in several forms, e.g., based on the type of information recalled, it can be defined as verbal and nonverbal. Discrimination of these two types of memory has special importance, because they involve different brain hemispheres. 

In another categorization, memory is also divided into three types, including sensory memory, short-term memory and long-term memory. Short-term memory includes WM and determining boundaries between short-term memory and WM is controversial. WM is the ability to maintain and process information simultaneously during the performance of a cognitive task. It is considered a central construction in cognitive psychology, besides plays an important role in scholastic activities like language comprehension (6). WM capacity is a predictor of performance on other cognitive tasks such as reading comprehension, reasoning, problem solving and executive function such as scheduling, organizing, strategizing, paying attention to and remembering details in daily life (6-8).

Considering the relationship between general cognitive development and proper learning process, several studies in recent years target many aspects of learning and try to find new factors and approaches effective in promoting individual learning (9-12). Both intelligence and memory have WM in common, so interaction and communication between WM and intelligence is implicated in learning.

The role of intelligence as a mental ability which supports many functions of the brain in learning and academic achievement has been debated (13, 14). Intelligence is considered as the most important factor of success, but is not the only factor in success (2, 13, 15-17). On one hand the role of intelligence is overshadowed by other cognitive factors such as WM, attention, and motivation, e.g., in some studies WM is determined as an underlying factor for learning disabilities (18, 19). On the other hand, WM capacity can be improved and appropriate exercises can help increasing capacity and improving the academic situation. For example, WM training can effectively improve ADHD and other cognition and Learning Disabilities (7, 8). 

WM digit span is a part of Wechsler WM test (IVth version) and includes two sub tests, Forward Digit Span (FDS) and Backward Digit Span (BDS). An easy test provides important information when evaluating working memory. One of the most popular tests used in evaluating mental abilities in Iran is the 4th edition of Wechsler intelligence test. Thereby most of the schools in Iran administer this test when registering students and record the IQ in their medical history. Subsequently students are classified into five different groups according to the Wechsler classification. 

In memory evaluation, an important point is that different stimulus used varies according to the type of memory being measured. Due to the different types of memory, the form of stimulus used to evaluate the content and manner of presentation and the responses vary and can involve different parts of the brain. Rey Auditory Verbal Learning Test (RAVLT) (20) is a well-recognized measure of a person’s ability to encode, combine, store and recover verbal information in different stages of immediate memory. Therefore, the effect of interference stimulus, delayed memory and recognition are evaluated with this assessment tool. It is translated and validated in multiple languages, including Finnish, Spanish, Hebrew, Chinese, German, Dutch, Greek and Persian (21). 

While the RAVLT in different articles among different population has been a sensitive test of verbal learning and memory, it was the tool to evaluate relationship between memory aspects and learning. It has been a sensitive instrument to measure the impact of intelligence in the learning process.

In the current study, considering students of two typical and gifted schools, evaluation of different stages of RAVLT and Digit Span was conducted to compare

aspects of memory between two student groups and particularly highlight the role of WM in RAVLT stages results.

## Materials & Methods

**Participants**

The current cross sectional comparing study by nonprobability sampling, was performed on 148 male students aged 12-14 yr old divided into two groups to

include 75 students in typical school with mean age± SD of 13.01 and 0.81 yr and 73 gifted students with mean age± SD as13 and 0.81 yr. This study was conducted

over 4 months from February to May 2013 in Tehran, Iran

All participants were tested using the Wechsler test (based on a valid Persian version) and the results were recorded in the student’s health records. The subjects were categorized into two groups using the Wechsler intelligence scale classification. The gifted students included 73 students with IQs ranging between 110 and 130. The typical students included 75 students with IQs ranging between 90 and 110 (Table 1). 

All students had normal hearing, were right handed dominant, and were monolingual in the Persian language.

None of the subjects had a history of recurrent ear infections, head trauma, epilepsy, use of psychotropic medications, or neuropsychological disease. After

subjects were divided into groups according to the intelligence scale found in the student’s health records, the Persian version of Digit Span and Rey Auditory

Verbal Learning Test were performed.

This study was approved by the Ethics Committee of Shahid Beheshti University, Tehran, Iran. We utilized the index of central tendency of mean and standard deviation of the distribution to evaluate the relationship between IQ scale and the separate Rey stages using the Spearman correlation test (bidirectional). In addition, data analysis was done using statistical software SPSS 18th version (Chicago, IL, USA) in significance level of 0.05.

**Materials/Procedures**

**Digit Span;**

The digit span procedure was derived from the standardized administration of the digits forward and backward subtests of the digit span test, as described in the Wechsler Intelligence Scale for Children–Forth Edition (WISC-IV) manual. Memory performance was assessed by the experimenter reading aloud series of digits at the rate of one item per second, and the child was instructed to repeat them back immediately in the correct sequence without any discrete recall cue. Three trials were given at each list length, beginning with a list length of two items. If recall was correct on two or more of the three trials for each list length, the sequence length was increased by one item. In each span task, the change in the number of elements was signaled by the experimenter telling the child, ‘‘Now let’s try it with “n” numbers” (a cue that potentially warned the child

about the list length and signaled the end of a presented sequence of items). If the child failed more than one list in a list length, testing was discontinued.

**Rey auditory-verbal learning test**

Using the RAVL Test (a 15 noun-word list (list A) was read to the participants with a presentation rate of one word per second. The Persian version of the word list was the same as used in a previous study (21). After presentation of the 15 words the persons were requested to recall as many words as possible (participants were instructed that the order was not important). The procedure was repeated 5 times, and after each trial recall was recorded.

The five recall trials were summed into one score (trial 1–5). After 5 presentations of list A, an interference-list of 15 other nouns (list B) were read to the participants and they were asked to recall as many words as possible. 

Immediately after recall of list B, the participants were again asked to recall list A (short recall, A6). Delayed recall of list A was measured 30 min after the immediate recall (long recall, A7) (with no other verbal memory tests administered in this interval). Directly after long recall, A7, a recognition trial of 30 words containing the 15 words from list A and 15 distracter items were applied (10 distracter words were semantically or phonetically similar to the target words). Different versions of

measuring recognition on RAVLT exist; some using 50 words, others just 30 words (22). The total scores for (trial 1–5), short recall (A6), long recall (A7) and the number of correct responses in the recognition test were analyzed.

**Procedure**

First, all participants completed the case history and then the examiner explained the details of the following tests.

The FDS test and BDS were administered sequentially to subjects with intervals of 15 after the RAVLT was administered based on standard instruction. 

**Table 1:** Mean and SD of Digit Span and first five stages of RAVL tests in two groups of typical and gifted students in different ages

**Group **	**age (yr)**	**FDS**	**BDS**	**REY1**	**REY2**	**REY3**	**REY4**	**REY5**
** Gifted**	**12 Mean** ** N** ** Std. Deviation** ** Min -Max **	6.4167 24 1.10007 5-8	4.6667 24 0.86811 3-6	6.6667 24 1.46456 4-9	9.2917 24 1.87615 6-13	10.9583 24 1.48848 8-14	11.9583 24 1.60106 9-15	12.6250 24 1.81330 9-15
	**13 Mean** ** N** ** Std. Deviation** ** Min-Max **	6.5600 25 1.12101 4-8	4.4400 25 0.96090 2-6	6.6800 25 1.700984- 9	8.4800 25 1.93907 5-11	10.3200 25 1.70098 7-13	11.4000 25 1.68325 8-14	12.2800 25 1.81475 8-15
	**14 Mean** ** N** ** Std. Deviation** ** Min-Max**	7.1667 24 1.12932 5-9	5.1667 24 1.00722 3-7	7.4167 24 1.50121 4-10	9.1250 24 1.65010 6-12	11.0000 24 1.50362 8-13	12.3333 24 1.43456 9-15	12.7500 24 1.77544 10-15
	**Total Mean** ** N** ** Std. Deviation** ** Min- Max **	6.7123 73 1.14842 4-9	4.7534 73 0.98292 2-7	6.9178 73 1.57897 4-10	8.9589 73 1.83665 5-13	10.7534 73 1.57921 7-14	11.8904 73 1.60348 8-15	12.5479 73 1.78762 8-15
** Typical**	**12 Mean** ** N** ** Std. Deviation** ** Min- Max **	5.2500 24 0.98907 4-8	3.8750 24 0.79741 3-6	5.4583 24 1.47381 3-8	7.5833 24 1.81579 4-13	9.2083 24 1.66757 6-12	10.2917 24 1.54580 7-13	10.5417 24 2.06375 6-14
	**13 Mean** ** N** ** Std. Deviation** ** Min- Max**	5.4615 26 1.24035 4-8	3.5000 26 .0.86023 2-5	5.8846 26 1.24344 3-8	7.6538 26 1.49512 5-11	9.2308 26 1.60767 7-13	10.5385 26 1.60576 7-13	11.1538 26 1.73649 8-14
	**14** **Mean** **N** **Std. Deviation ** **Min- Max**	5.1200 25 1.05357 3-7	3.6000 25 0.70711 2-5	6.2000 25 0.95743 4-8	8.3200 25 1.54704 5-11	9.7200 25 1.48661 6-12	10.7600 25 1.42244 6-12	11.1600 25 1.43411 7-13
	**Total Mean** ** N** ** Std. Deviation** ** Min- Max**	5.2800 75 1.09742 3-8	3.6533 75 0.79684 2-6	5.8533 75 1.25949 3-8	7.8533 75 1.63321 4-13	9.3867 75 1.58450 6-13	10.5333 75 1.51865 6-13	10.9600 75 1.75869 6-14

**Fig 1:**
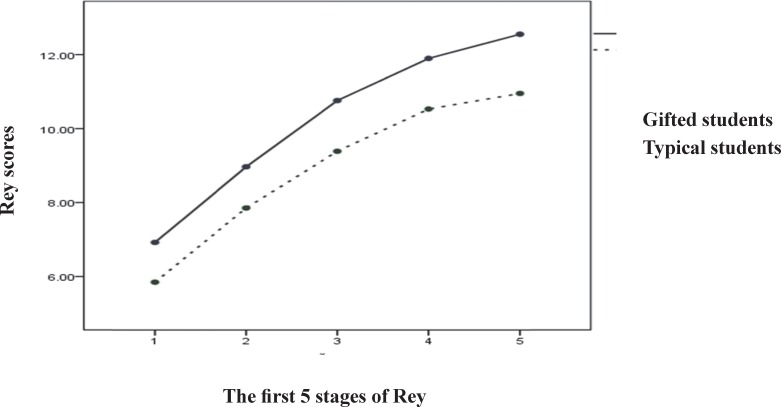
Comparing the results of first 5 stages of RAVLT among Typical and Gifted students

**Fig 2:**
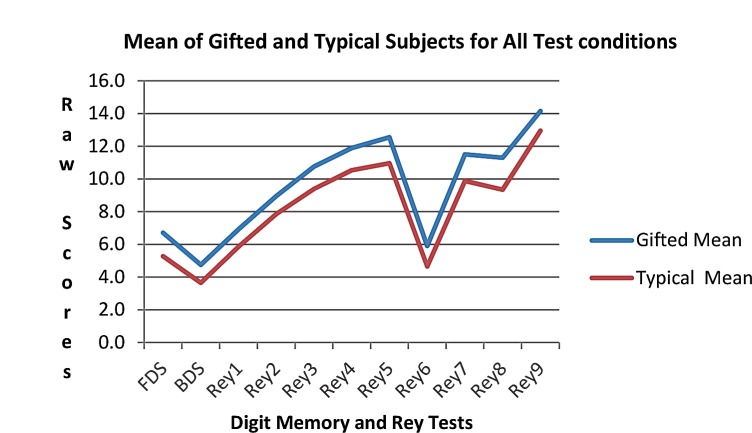
Comparison between Digit scores (FDS & BDS) and Rey stages scores (Rey 1 to Rey 9) among typical students and gifted students

**Table 2:** Mean and SD of last 4 stages of RAVL tests in two groups of typical and gifted students in different ages

**Group **	**Age (yr)**	**REY6**	**REY7**	**REY8**	**REY9**
** Gifted**	**12 Mean** ** N** ** Std. Deviation** ** Min -Max **	5.8750 24 1.75233 3-11	11.00 24 2.06419 7-14	10.5833 24 2.24416 6-14	14.1250 24 1.07592 11-15
	**13 Mean** ** N** ** Std. Deviation ** ** Min-Max **	5.8800 25 1.92180 3-9	11.1600 25 2.19241 7-15	11.3200 25 1.70098 8-14	14.2000 25 .76376 13-15
	**14 Mean** ** N** ** Std. Deviation** ** Min-Max**	5.9583 24 1.19707 4-8	12.3750 24 1.90680 8-15	12.0000 24 2.18692 7-15	14.1250 24 1.19100 11-15
	**Total Mean** ** N** ** Std. Deviation** ** Min- Max **	5.9041 73 1.63439 3-11	11.5068 73 2.12213 7-15	11.3014 73 2.10611 6-15	14.1507 73 1.00928 11-15
** Typical**	**12 Mean** ** N** ** Std. Deviation ** ** Min- Max **	4.3333 24 1.12932 2-6	9.8333 24 1.92617 6-14	9.6250 24 1.81330 6-13	12.9583 24 1.60106 8-15
	**13 Mean** ** N** ** Std. Deviation** ** Min- Max**	4.9615 26 1.45549 2.00 7.00 5.00	9.6154 26 1.69887 6-12	8.7308 26 1.82335 5-12	12.8462 26 1.71330 7-15
	**14** **Mean** **N** **Std. Deviation** **Min- Max**	4.6400 25 1.28712 2.00 7.00 5.00	10.2000 25 1.93649 4-13	9.7200 25 1.62070 6-12	13.0400 25 1.20692 11-15
	**Total Mean** ** N** ** Std. Deviation** ** Min- Max**	4.6533 75 1.30998 2-7	9.8800 75 1.84508 4-14	9.3467 75 1.78956 5-13	12.9467 75 1.50578 7-15

**Table 3:** Two-tailed spearman correlations between the subject’s IQ and results of the FDS, BDS, and stages of the REY tests.

	**FDS**	**BDS**	**Rey1**	**Rey2**	**Rey3**	**Rey4**	**Rey5**	**Rey6**	**Rey7**	**Rey8**	**Rey9**
**IQ Correlation** **Coefficient**	0.630	0.596	0.472	0.478	0.527	0.533	0.561	0.458	0.495	0.559	0.595
**Sig**	0.000	0.000	0.000	0.000	0.000	0.000	0.000	0.000	0.000	0.000	0.000
**N**	148	148	148	148	148	148	148	148	148	148	148

**Table 4:** Results of Pearson Product Moment Correlations between Digit Span & RAVLT Stages for all subjects.

	**BDS**	**Rey1**	**Rey2**	**Rey3**	**Rey4**	**Rey5**	**Rey6**	**Rey7**	**Rey8**	**Rey9**
**FDS** **Sig**	0.759 0.000	0.636 0.000	0.551 0.000	0.610 0.000	0.586 0.000	0.542 0.000	0.538 0.000	0.639 0.000	0.605 0.000	0.481 0.000
**BDS** **Sig**		0.552 0.000	0.473 0.000	0.551 0.000	0.610 0.000	0.586 0.000	0.542 0.000	0.586 0.000	0.542 0.000	0.538 0.000
**Rey1** **Sig**			0.771 0.000	0.586 0.000	0.542 0.000	0.538 0.000	0.542 0.000	0.586 0.000	0.542 0.000	0.538 0.000
**Rey2** **Sig**				0.765 0.000	0.542 0.000	0.586 0.000	0.586 0.000	0.610 0.000	0.605 0.000	0.481 0.000
**Rey3** **Sig**					0.807 0.000	0.542 0.000	0.538 0.000	0.586 0.000	0.542 0.000	0.538 0.000
**Rey4** **Sig**						0.795 0.000	0.542 0.000	0.586 0.000	0.542 0.000	0.586 0.000
**Rey5** **Sig**							0.564 0.000	0.586 0.000	0.542 0.000	0.538 0.000
**Rey6** **Sig**								0.497 0.000	0.538 0.000	0.542 0.000
**Rey7** **Sig**									0.826 0.000	0.586 0.000
**Rey8** **Sig**										0.598 0.000

## Results

Participants were divided into two groups of typical and gifted students based on Wechsler classification. A Chisquare test was performed and a significant difference was found between different IQs among students in the two groups, X2(4) =116, P=0.000. While there was no significant difference between different ages. X2 (2) =0.013 ، P=0.994. 

The mean, standard deviation score, number and minimum- maximum scores of students for the Digit

span and first 5 stages of RAVLT are shown in Table 1.

Accordingly, FDS had a greater mean score comparing with BDS and had close score to the first stage of Rey (Table 1).

The first five stages were five repetitions of the same list of words referred to as a learning curve. Figure 1 illustrates the growth in scores from the first stage up to the fifth.

There is also detailed information of the last four stages in the Table 2. Moreover, the Mean score for all tests is shown in Figure 2. As it can be seen in the Figure 1 and 2 and Tables 1 and 2 gifted students obtained higher scores than typical students in both FDS and BDS and all 9 stages of RAVLT comparing with typical students and this difference was shown significant by t-test (P<0.001). While one way ANOVA analysis did not show significant difference between different ages (P> 0.05) the 14 yr old students in both groups had the highest score.

To evaluate difference in performance of RAVLT stages, Repeated Measurement ANOVA was taken

and the difference between the stages was significant (P< 0.001) and these stages were different one by one.

This difference of group performance was significant at approximately the same level across test conditions. The gifted students had higher scores compared with typical students (P< 0.001). Figure 2 also shows that the Rey 6 test was substantially lower than adjacent conditions and the difference was significant (P< 0.001). The ninth stage had the highest score. This stage involves recognition of the repeated list words containing 50 written words.

Table 3 shows the two-tailed Spearman correlations between all subject’s IQ and results of the FDS, BDS, and stages of the Rey tests. There was a significant positive correlation among these measures with (P<0001). Results of Pearson Product Moment correlations across subjects are shown in Table 4. There was a high correlation between FDS and the first stage of RAVLT and also the high correlation between BDS and seventh stage of RAVLT. Furthermore since the mean scores for all groups are parallel, there was not separate correlations shown for the gifted and the typical subjects and the subjects are all grouped.

## Discussion

The current study evaluated digit memory of normal hearing gifted and typical adolescent students using a WM digit span test. Gifted students obtained higher scores in both FDS and BDS compared with typical students. Furthermore, the FDS score was higher than BDS in both groups. FDS and BDS are separately interpreted; FDS evaluates Short Term Memory (STM) and BDS because of more complicated mental processing evaluates WM.

There are several studies on the relationship between memory and intelligence conducted on adults but few of them studied this relationship among children and teenagers (15). In adults, WM was the closest concept to intelligence evaluated, in some of these studies, close relationship was seen between WM and intelligence (24). In a study, 200 children aged 6 to 16 yr old used the Digit Span test of Italian version of Wechsler test.

Accordingly, WM was evaluated as fourth subscale of Wechsler test, this part itself has three subscales: 1).

Digit span (FDS and BDS) 2) Letter Number Sequence (LNS) and 3) Arithmetic (AR). It was indicated that the tests, which need more brain challenges in comparison with the tests, and need less challenges have the higher relationship with intelligence (15). In other studies, also the relation between different intelligence types and WM components was confirmed (8, 25-27).

WM has two essential and basic components; STS and non-storage components. Short term storage has the most important role in relation between intelligence and WM (28). On the other hand, there are some studies, which do not confirm the relation between intelligence and WM. For example, these two items as two separate and independent mental structures were reported (2, 7).

Researchers found low to average correlation between intelligence and WM (29,30). Colom valuated the effects of WM trainings on WM and intelligence, and indicated that those trains improved the WM but did not have effect on intelligence (28). The difference among these studies firstly can be attributed to the different WM tests and consequently different aspects of WM, which were evaluated. Indeed, Wechsler WM test is the best way to show that different aspects of WM have different relationship with intelligence (17). Secondly, it is due to the difference between the simple and complicated tasks, of higher resistance against interferences in first group.

The considerable point is that 6 and 7 stages had lower scores than the first five stages which may be due to the negative effects of interferences in both groups’ results.

In fact, in 6 and 7 stages the brain involves resisting against interferences and this itself results in lower score in these stages comparing with 1 to 5 stages. This result is confirmed earlier (35-39). Therefore VI and VII stages have a closer meaning to WM rather than STM (33-40).

VI stage had the lower score than I among all subjects but gifted subjects had higher score in VII stage comparing with normal subjects while Geffen accounted superior effect in VII stage among intellectual subjects (30). This paradox possibly is due to different aging group, which was evaluated.

Finally, in 7 and 9 stages also gifted students had better performance than typical students which indicates better Long Term Memory in this group. In a study, the intelligence and educational level effect on every stages of RAVLT were evaluated and concluded that the more intelligence result in higher score in RAVLT, additionally they assumed that this effect for different aging group varied (31). They relate this finding to higher processing speed and more attention and recognition ability in intellectual students. In some other studies, intelligence was effective in the VIII and IX stages.

For instance in one study there was not any significant relationship in last three stages (32) or another study reported intelligence effect just in VIII stage or the other one reported no effect in 8 and 9 stages (34, 40). These conclusions might be due to limited intelligence range in those studies and the other point is that there was not any gifted subject in their studies while in current study there was intelligence that was more superior.

When comparing Digit test and Rey test subscales, some interesting results can be concluded. First it should be considered based on a study lateral premotor cortex is main central language center, although there are some differences. Indeed rostral and dorsal parts are showed significantly greater activity during the numeral task than the word task whereas its caudal part (PMdc) was similarly active during the two tasks (41). Analyzing the correlation between Digit Span subscales and the RAVLT stages, FDS and BDS had the highest correlation with I and VII stages of RAVLT respectively, and both of them had the lowest correlation with ninth stage.

There are several studies, which compared different memory tests (21-32), but only in one study, Rey and digit span are compared and the same result was seen (23), conducted among children with learning disability, which found high correlation between RAVLT stage I, and FDS and between RAVLT stage VII and BDS. As entioned above FDS and BDS evaluate STM and M respectively. There are evidences to assume the first stage of RAVLT as the ability of STM, so it can be rationalized that there are high correlation between FDS and first stage of RAVLT and poor relationship between BDS and first stage.

Since in VII stage comparing with the first has more challenge to resist against interferences is more close to WM and has higher correlation with BDS. Additionally BDS and Rey VII had lower score than FDS and Rey I. In a literature review study comparing STM and WM, assume WM due to higher and more complicated cognitive functions and pointed out that WM capacity is lower than STM (10). Researchers evaluated WM capacity with BDS and comparing with FDS and confirmed the previous results (25, 39). 

**In conclusion**, there were significant relationship between intelligence and memory capacity but slope of learning with repetition was the same for two groups; moreover, it showed that FDS and BDS were inadequate to indicate all aspects of memory and capacity of learning. However, it can be used as a supplementary tool for screening subjects with learning problems.
